# Significance of druggable targets (PD-L1, KRAS, BRAF, PIK3CA, MSI, and HPV) on curatively resected esophageal squamous cell carcinoma

**DOI:** 10.1186/s13000-020-01045-4

**Published:** 2020-10-14

**Authors:** Hong Kyu Lee, Mi Jung Kwon, Yong Joon Ra, Hee Sung Lee, Hyoung Soo Kim, Eun Sook Nam, Seong Jin Cho, Hye-Rim Park, Soo Kee Min, Jinwon Seo, Ji-Young Choe, Kyueng-Whan Min, So Young Kang

**Affiliations:** 1 Department of Thoracic and Cardiovascular Surgery, Hallym University Sacred Heart Hospital, Hallym University College of Medicine, Anyang, Republic of Korea; 2 Department of Pathology, Hallym University Sacred Heart Hospital, Hallym University College of Medicine, Gwanpyeong-ro 170 beon-gil 22, Dongan-gu, Anyang-si, Gyeonggi-do 14068 Republic of Korea; 3 Department of Thoracic and Cardiovascular Surgery, Hallym University Dongtan Sacred Heart Hospital, Hallym University Medical Center, Hwaseong-si, Gyeonggi-do Republic of Korea; 4 Department of Pathology, Kangdong Sacred Heart Hospital, Hallym University College of Medicine, Chuncheon, Republic of Korea; 5 Department of Pathology, Hanyang University Guri Hospital, Hanyang University College of Medicine, Seoul, Republic of Korea; 6 Department of Pathology, Samsung Medical Center, Sungkyunkwan University College of Medicine, Seoul, Republic of Korea

**Keywords:** Programmed death-ligand 1, Esophagus, Squamous cell carcinoma, Microsatellite instability, Human papillomavirus, *PIK3CA*

## Abstract

**Background:**

Esophageal squamous cell carcinoma (ESCC) still remains intractable disease with few therapeutic options. Programmed death-ligand 1 (PD-L1), which is essential for immune evasion, is involved in the pathogenesis of ESCC and thus is a potential therapeutic target. *PIK3CA*, *KRAS*, and *BRAF* mutations, microsatellite instability (MSI) caused by deficient mismatch repair (dMMR), and human papillomavirus (HPV) can potentially upregulate PD-L1 expression, which might contribute to the clinical outcome of patients with ESCC.

**Methods:**

We investigated the significance of the present druggable markers [PD-L1, *PIK3CA*, *KRAS*, and *BRAF* mutations, MSI caused by deficient dMMR, and HPV] in 64 curatively resected ESCCs, using immunohistochemistry (PD-L1 and MMR protein expression), direct sequencing (*KRAS*, *BRAF*, and *PIK3CA* mutations), real-time PCR (HPV infection), and MSI using quasi-monomorphic markers.

**Results:**

PD-L1 expression, *PIK3CA* mutation, and MSI/dMMR were detected in 35.9, 12.5, and 17.2% of ESCCs, respectively. HPV was rarely detected (1.6%) (high-risk HPV68), whereas *KRAS* and *BRAF* mutations were not detected in ESCCs. PD-L1-positive tumors were not correlated with *PIK3CA* mutation or MSI/dMMR (all *P* > 0.05). PD-L1, *PIK3CA* mutation, and MSI/dMMR characterized the patients associated with light smoking, female and younger age, and younger age and well-differentiated tumors, respectively (all *P* < 0.05). In multivariate analysis, only PD-L1-positivity was an independent favorable prognostic factor for overall survival (OS) and disease-free survival (DFS) (*P* = 0.023, *P* = 0.014). In the PD-L1-negative ESCCs, *PIK3CA* mutation had a poor prognostic impact on both OS and DFS (*P* = 0.006, *P* = 0.002).

**Conclusions:**

*PIK3CA* mutation may be an alternative prognostic biomarker in PD-L1-negative curatively resected ESCCs that can be optional to identify high-risk patients with worse clinical outcome who require more intensive therapy and follow-up.

## Introduction

Globally, esophageal cancer is the seventh most prevalent cancer and the sixth most common cause of cancer-related mortality, representing one of the most aggressive malignant tumors that are very difficult to treat [1]. The prognosis of patients with ESCC is poor with a five-year survival rate of 15–30% even for patients who undergo complete resection [2]. Among the Korean population, esophageal cancer is the eighth most common cause of cancer-related mortality [3]. The incidence and mortality rates are high among Korean males with esophageal squamous cell carcinoma (ESCC), which is the most common histological form of esophageal cancer in Korea [3]. Although there have been rapid advances in surgical techniques and multimodal chemoradiation therapies for other cancers, ESCCs still remain intractable disease with few therapeutic options. The risk factors for ESCCs are also less established in the Korean population.

There are no established prognostic and predictive markers as well as druggable markers for ESCCs as there is limited knowledge on the targetable genetic abnormalities in ESCC [4, 5]. Recently, cancer immunotherapy, which exploits the patient immune system to attack cancer cells, has been gaining attention for treating esophageal cancers [6]. One of the major hallmarks of cancer is the evasion of the immune system, which enables the cancer cells to escape the attack from immune cells [7]. The cancer cells can express several immune inhibitory signaling proteins, which induce immune cell dysfunction and apoptosis [7]. Programmed death-ligand-1 (PD-L1), an immune inhibitory protein, binds to programmed death-1 (PD-1) expressed on the tumor-infiltrating lymphocytes (TILs), such as T-cells, B-cells, dendritic cells, and natural killer T-cells to suppress anti-cancer immunity and enable neoplastic growth [7]. Recent studies have demonstrated that PD-L1 expression was detected in 30–45% of ESCCs and that PD-1 blockade was effective in only a subset of patients with PD-L1-positive esophageal cancer [6]. This suggested that the PD-1/PD-L1 axis is highly related to ESCCs and that the identification of additional biomarkers can aid in predicting the clinical response of patients with ESCC. As PD-L1 expression in ESCC has clinical importance, it is important to elucidate the mechanisms underlying the regulation of PD-L1 expression.

The Cancer Genome Atlas (TCGA) reported that *PIK3CA* gene alterations and related genetic alterations, which were evaluated by exome sequencing, activate the phosphatidylinositol 3-kinase (PI3K) pathway in 24% of ESCCs that can be targeted by small molecules [8]. The PD-L1/PD-1 axis is reported to be driven by the oncogenic activation of the signaling pathways, such as the mitogen-activated protein kinase (MAPK) and PI3K pathways. Additionally, this axis is regulated by other factors in the tumor microenvironment, including microsatellite instability (MSI) caused by deficient mismatch repair (dMMR) and human papillomavirus (HPV) [9–13]. However, MSI/dMMR, *RAS*/*BRAF*/*PIK3CA* mutations, and HPV status have been rarely investigated in esophageal cancers. PD-L1 blockade is used to treat gastrointestinal cancers exhibiting MSI/dMMR, while a combination of targeted therapies is used to treated gastrointestinal cancers with *RAS*/*BRAF*/*PIK3CA* mutation [10, 13]. Thus, there is a need for a comprehensive evaluation in esophageal cancers combining the present druggable and targetable biomarkers. In this study, we investigated PD-L1 expression in association with *PIK3CA, KRAS*, and *BRAF* mutations, MSI/dMMR status, and HPV infection to determine their prognostic relevance for curatively resected ESCCs.

## Patients and methods

The patient medical charts and pathology reports were retrieved for identifying surgically resected and pathologically confirmed ESCC cases. In total, 78 ESCC cases were obtained between 2000 and 2018. Based on the hematoxylin and eosin (H&E) slide review and chart review, only patients with primary esophageal cancers who were chemotherapy- or radiation therapy-naive at the time of the surgery, and whose formalin-fixed, paraffin-embedded (FFPE) tumor tissue blocks and complete medical charts were available were included in this study. Nine patients treated with preoperative chemotherapy or radiotherapy that might induce any different bias in the study and five patients unavailable for specimens were excluded. In total, 64 patients with ESCC who underwent radical esophagectomy (Ivor-Lewis operation) with standard lymphadenectomy as the initial definitive treatment were included in the study.

The clinical characteristics and follow-up data were retrospectively obtained from the medical records, pathology report files, and radiological study results. Based on the clinician’s judgment, the type of esophageal resection was determined and the selected patients were offered adjuvant chemotherapy with or without radiation therapy. The radiation doses ranged from 5000 cGy to 5400 cGy (23 dose fractions) over a period of 8 weeks. The patients underwent fluoropyrimidine−/taxene-based chemotherapy. Chemotherapy was prescribed based on the performance statuses, comorbidities, and toxicity profiles of individual patients.

Smoking history was measured in packs per year and the patients were classified into 2 categories using 20 packs/year as the cut-off value with heavy smoking defined as > 20 packs/year [14]. Alcohol consumption status was divided into 2 categories using 14 drinks/week as the cut-off value and heavy alcohol consumption was defined as > 14 drinks/week [14].

The pathology reports and histological slides were reviewed and were re-evaluated by an experienced gastrointestinal pathologist (MJK). The diagnosis and histological differentiation were evaluated according to the World Health Organization classification [15]. The stage of the esophageal carcinomas was determined based on the American Joint Committee on Cancer staging system (Eighth edition) [14].

The degree of infiltration of TILs was evaluated from the H&E-stained slides and scored from 0 to 3 as follows: 0, none; 1+, focal infiltration with more than mild staining intensity; 2+, moderate infiltration with more than mild staining intensity; 3+, diffuse infiltration with more than mild staining intensity.

The study protocol was approved by Hallym University Sacred Heart Hospital Institutional Review Board (IRB No. 2019–03–014-001) and performed in accordance with the relevant guidelines and regulations (Declaration of Helsinki). Informed consent was obtained from the patients and from the next of kin (deceased patients) before enrollment in the study.

### Immunohistochemistry and microsatellite status determination

Immunohistochemical staining was performed on 4-μm thick tissue microarray (TMA) sections using the BenchMark XT automated immunostainer system (Ventana Medical Systems, Inc., Tucson, AZ, USA), according to the manufacturer’s instructions. Using a TMA manufacture tool (Quick-Ray™; Unitma, Seoul, South Korea), TMAs were constructed from two 3.0-mm cores of tumor tissue from the representative areas of each case after carefully reviewing all H&E-stained slides. The primary antibody used in this analysis was anti-PD-L1 (rabbit anti-human PD-L1 monoclonal, 1:25, clone SP142; Ventana). PD-L1 expression was evaluated based on the proportion of membranous staining in the tumor cells. The expression was scored as follows: 0 for < 5% of tumor cells, 1+ for 5–10%, 2+ for 10–50%, and 3+ for > 50% of tumor cells. Consistent with several previous published reports, an IHC score of ≥1+ was considered positive [12, 16]. To evaluate the expression of MMR proteins, the following primary antibodies were used in this analysis: anti-MLH1 (pre-diluted; Ventana Medical Systems), anti-MLH2 (1:300; Cell Marque, Rocklin, CA, USA), anti-MSH6 (1:200; Cell Marque), and anti-PMS2 (pre-diluted; Ventana Medical Systems) antibodies.

MSI status in the tumor was analyzed by immunohistochemistry of MMR proteins and by multiplex polymerase chain reaction (PCR) for five quasi-monomorphic mononucleotide repeat markers (BAT25, BAT26, NR21, NR24, and NR27). The MSI/dMMR tumors were defined as those exhibiting loss of expression of one or more MMR proteins or exhibiting high-level MSI (MSI-H), which was determined by PCR [17, 18]. Microsatellite-stable/proficient MMR (MSS/pMMR) tumors exhibited intact MMR protein expression and/or MSS or low-level MSI (MSI-L) status.

### Mutation and HPV detection analyses

Genomic DNA was extracted from 10-μm thick sections of 10% neutral manually dissected FFPE tumor tissue using the Maxwell® 16 FFPE Purification Kit for DNA (Promega, USA). The mutations in the *KRAS* (exon 2 and 3), *BRAF* (exon 14), and *PIK3CA* (exon 9 and 20) genes were analyzed by directional sequencing of PCR fragments amplified from the genomic DNA as previously described [12]. All sequences were confirmed in duplicate for replicate amplification reactions.

The HPV status was determined using the PANA RealTyper™ HPV Kit (PANAGENE, Daejeon, South Korea), according to the manufacturer’s instructions. This kit, which is approved for clinical use in Korea, detects 40 HPV genotypes, including 20 high-risk genotypes (16, 18, 26, 31, 33, 35, 39, 45, 51, 52, 53, 56, 58, 59, 66, 68, 69, 70, 73, and 82), 2 low-risk genotypes (6 and 11), and 18 other genotypes (30, 32, 34, 40, 42, 43, 44, 54, 55, 61, 62, 67, 74, 81, 83, 84, 87, and 90).

### Statistical analysis

The clinical and pathological parameters are represented as mean ± standard deviation for continuous variables and as frequency for categorical variables. The categorical variables were analyzed using the chi-squared (χ^2^) test or two-sided Fisher’s exact test. Survival analyses were performed using the Kaplan-Meier method. The survival curves were compared using the log-rank test. Overall survival (OS) was defined as the interval from the first day of surgery until death or the end of the follow-up period. Disease-free survival (DFS) was defined as the interval from the first day of surgery until tumor progression, death, or end of the follow-up period. We also used the Cox proportional hazards model for the univariate and multivariate analyses of OS and DFS. OS and DFS were analyzed until February 2019. All statistical analyses were performed in the SPSS statistical analysis software (v18; SPSS, Chicago, IL, USA). The difference was considered statistically significant when the *P*-value was less than 0.05.

## Results

### Patient demographic characteristics

Of the 64 patients, 60 (93.8%) were male and 4 (6.2%) were female, with the median age at diagnosis of 63 years (range 46–84 years) (Table 1). Approximately half of the patients were either heavy smokers (35/64, 54.7%) or heavy alcohol drinkers (32/64, 50.0%), while common smokers and alcohol drinkers accounted for 35.9% (23/64). Middle esophageal region (48.4%) was dominant compared to lower (29.7%) or upper (21.9%) regions. Locally advanced cancers (pT2-pT4: 57.8%) and early cancers (pT1: 42.2%) were roughly evenly distributed, while synchronous lymph node metastases (pN+) were detected in 25 cases (39.1%). However, no synchronous distant metastases (pM1: 0%) were identified. The follow-up period ranged from 3 to 123 months. At the last follow-up appointment, 26 patients (40.6%) were alive, 40 patients (62.5%) had tumor relapse, and 38 patients (59.4%) had died. The 1-year, 3-year, and 5-year OS rates were 64.4, 34.7, and 23.9%, respectively.

**Table 1:** Baseline patient characteristics of patients with esophageal squamous cell carcinoma

Clinicopathological	Total
variables	*N* = 64 (%)
Sex	
Male/Female	60 (93.8)/4 (6.2)
Age, median years	63.5
Smoking (pack-yrs)	
Light/Heavy	29 (45.3)/35 (54.7)
Alcohol (drink/week)	
Light/Heavy	32 (50.0)/32 (50.0)
Location	
Upper/Middle/Lower	14 (21.9)/31 (48.4)/19 (29.7)
Tumor size, median (range, mm)	36.5 (8–105)
Differentiation	
Well/Moderate/Poor	26 (40.6)/26 (40.6)/12 (18.8)
pT category	
T1/T2/T3/T4	27 (42.2)/9 (14.1)/27 (42.2)/1 (1.5)
pN category	
N0/N1/N2/N3	39 (60.9)/14 (21.9)/7 (10.9)/4 (6.3)
pM category	
M0/M1	64 (100)/0 (0)
AJCC stage (8th)	
IA/IB	3 (4.7)/23 (35.9)
IIA/IIB	9 (14.1)/5 (7.8)
IIIA/IIIB	4 (6.3)/15 (23.4)
IVA	5 (7.8)
Treatment	
Surgery alone	47 (73.4)
Surgery and CT	7 (10.9)
Surgery and RT	1 (1.6)
Surgery and CCRT	9 (14.1)
Resection margin status	
Negative (R0)/Positive (R1)	57 (89.1)/7 (10.9)
Recurrence	
No/Yes	24 (37.5)/40 (62.5)
Survival status (at follow-up)	
Alive/Dead	26 (40.6)/38 (59.4)
Family history of malignancy	2 (3.1)
Metachronous/synchronous malignancy	17 (26.6)

*CT* chemotherapy, *RT* radiotherapy, *CCRT* concurrent chemoradiation therapy

Light smoking was associated with the N0 category (*P* = 0.040) and high TIL (*P* = 0.018). Heavy alcohol drinking was associated with male patients (*P* = 0.024). Other clinical or pathological parameters were not associated with smoking or alcohol consumption (Supplementary Table 1).

### PD-L1 expression, *PIK3CA* mutation, MSI, and their clinicopathological correlation

The correlation between clinicopathologic characteristics of the patients and PD-L1 expression, *PIK3CA* mutation, and MSI status was evaluated (Table 2).

**Table 2:** Clinicopathologic correlations of PD-L1 expression, *PIK3CA* mutation, and MSI/dMMR status

		PD-L1 expression		*P*	*PIK3CA*		*P*	MSI/dMMR		*P*
	Total	Positive	Negative		MT	WT		Positive	Negative	
	N = 64 (%)	*n* = 23 (35.9%)	*n* = 41 (64.1%)		*n* = 8 (12.5%)	*n* = 56 (87.5%)		*n* = 11 (17.2%)	*n* = 53 (82.8%)	
Sex				0.545			**0.019**			1.000
Male	60 (93.8)	21 (91.3)	39 (95.1)		6 (75.0)	54 (96.4)		11 (100)	49 (92.5)	
Female	4 (6.2)	2 (8.7)	2 (4.9)		2 (25.0)	2 (3.6)		0 (0.0)	4 (7.5)	
Age (years)				0.291			**0.043**			**0.039**
≤ 60	26 (40.6)	7 (30.4)	19 (46.3)		6 (75.0)	20 (35.7)		8 (72.7)	18 (34.0)	
> 60	38 (59.4)	16 (69.6)	22 (53.7)		2 (25.0)	36 (64.3)		3 (27.3)	35 (66.0)	
Smoking				**0.048**			0.850			0.322
Light	34 (53.1)	16 (69.6)	18 (4.9)		4 (50.0)	30 (53.6)		4 (36.4)	30 (56.6)	
Heavy	30 (46.9)	7 (30.4)	23 (56.1)		4 (50.0)	26 (46.4)		7 (63.6)	23 (43.4)	
Alcohol				0.728			0.564			0.176
Light	26 (40.6)	10 (43.5)	16 (39.0)		4 (50.0)	22 (39.3)		2 (18.2)	24 (45.3)	
Heavy	38 (59.4)	13 (56.5)	25 (61.0)		4 (50.0)	34 (60.7)		9 (81.8)	29 (54.7)	
Location				0.216			0.179			1.000
Upper/Middle	45 (70.3)	14 (60.9)	31 (48.4)		4 (50.0)	41 (73.2)		8 (72.7)	37 (69.8)	
Lower	19 (29.7)	9 (39.1)	10 (15.6)		4 (50.0)	15 (26.8)		3 (27.3)	16 (30.2)	
Differentiation				0.291			0.253			**0.039**
WD	26 (40.6)	7 (30.4)	19 (46.3)		5 (62.5)	21 (37.5)		8 (72.7)	18 (34.0)	
MD/PD	38 (59.4)	16 (69.6)	22 (53.7)		3 (37.5)	35 (62.5)		3 (27.3)	35 (66.0)	
T category				0.622			0.282			0.428
T1-T2	36 (56.2)	12 (52.2)	24 (58.5)		3 (37.5)	33 (58.9)		5 (45.5)	31 (58.5)	
T3-T4	28 (43.8)	11 (47.8)	17 (41.5)		5 (62.5)	23 (41.1)		6 (54.5)	22 (41.5)	
N category				0.282			0.498			0.247
N0	39 (60.9)	12 (52.2)	27 (65.9)		4 (50.0)	35 (62.5)		5 (45.5)	34 (64.2)	
N1–3	25 (39.1)	11 (47.8)	14 (34.1)		4 (50.0)	21 (37.5)		6 (54.5)	19 (35.8)	
AJCC stage				0.459			0.435			0.199
I-II	40 (62.5)	13 (56.5)	27 (65.9)		4 (50.0)	36 (64.3)		5 (45.5)	35 (66.0)	
III-IV	24 (37.5)	10 (43.5)	14 (34.1)		4 (50.0)	20 (35.7)		6 (54.5)	18 (34.0)	
LI				0.291			0.456			1.000
Absent	26 (40.6)	7 (30.4)	19 (46.3)		2 (25.0)	24 (42.9)		4 (36.4)	22 (41.5)	
Present	38 (59.4)	16 (69.6)	22 (53.7)		6 (75.0)	32 (57.1)		7 (63.6)	31 (58.5)	
VI				0.648			0.683			1.000
Absent	44 (68.8)	15 (65.2)	29 (70.7)		5 (62.5)	39 (69.6)		8 (72.7)	36 (67.9)	
Present	20 (31.2)	8 (34.8)	12 (29.3)		3 (37.5)	17 (30.4)		3 (27.3)	17 (32.1)	
PI				0.755			0.196			1.000
Absent	51 (79.7)	19 (82.6)	32 (78.0)		5 (62.5)	46 (82.1)		9 (81.8)	42 (79.2)	
Present	13 (20.3)	4 (17.4)	9 (22.0)		3 (37.5)	10 (17.9)		2 (18.2)	11 (20.8)	
Skip lesion				0.345			0.673			0.431
Absent	50 (78.1)	20 (87.0)	30 (73.2)		7 (87.5)	43 (76.8)		10 (90.9)	40 (75.5)	
Present	14 (21.9)	3 (13.0)	11 (26.8)		1 (12.5)	13 (23.2)		1 (0.1)	13 (24.5)	
Dysplasia				0.610			0.060			0.058
Absent	36 (56.2)	14 (60.9)	22 (53.7)		7 (87.5)	29 (51.8)		9 (81.8)	27 (50.9)	
Present	28 (43.8)	9 (39.1)	19 (46.3)		1 (12.5)	27 (48.2)		2 (18.2)	26 (49.1)	
TIL density				0.170			0.320			0.395
Low	22 (34.4)	5 (21.7)	17 (41.5)		4 (50.0)	18 (32.1)		5 (45.5)	17 (32.1)	
High	42 (65.6)	18 (78.3)	24 (58.5)		4 (50.0)	38 (67.9)		6 (54.5)	36 (67.9)	
HPV				1.000			1.000			1.000
Negative	63 (98.4)	23 (100)	40 (97.6)		8 (100)	55 (98.2)		11 (100)	52 (98.1)	
Positive	1 (1.6)	0 (0.0)	1 (2.4)		0 (0.0)	1 (1.8)		0 (0.0)	1 (1.9)	
MSI/MMR				0.732			0.531			–
MSS/pMMR	53 (82.8)	20 (87.0)	33 (80.5)		6 (75.0)	47 (83.9)		–	–	
MSI/dMMR	11 (17.2)	3 (13.0)	8 (19.5)		2 (25.0)	9 (16.1)		–	–	
*PIK3CA* status				0.700						
Wildtype	56 (87.5)	21 (91.3)	35 (85.4)		–	–		–	–	
Mutated	8 (12.5)	2 (8.7)	6 (14.6)		–	–		–	–	

*ESCC* esophageal squamous cell carcinoma, *PD-L1* programmed death ligand-1, *MT* mutated, *WT* wild type, *MSI* microsatellite instability, *pMMR* patent mismatch repair, *dMMR* deficient mismatch repair, *AJCC* Amedican Joint Committee on Cancer 8th edition, *WD* well-differentiation, *MD* moderately-differentiation, *PD* poorly-differentiation, *LI* lymphatic invasion, *VI* vascular invasion, *PI* perineural invasion, *TIL* tumor-infiltrating lymphocyte, *HPV* human papillomavirus

**Fig. 1:**
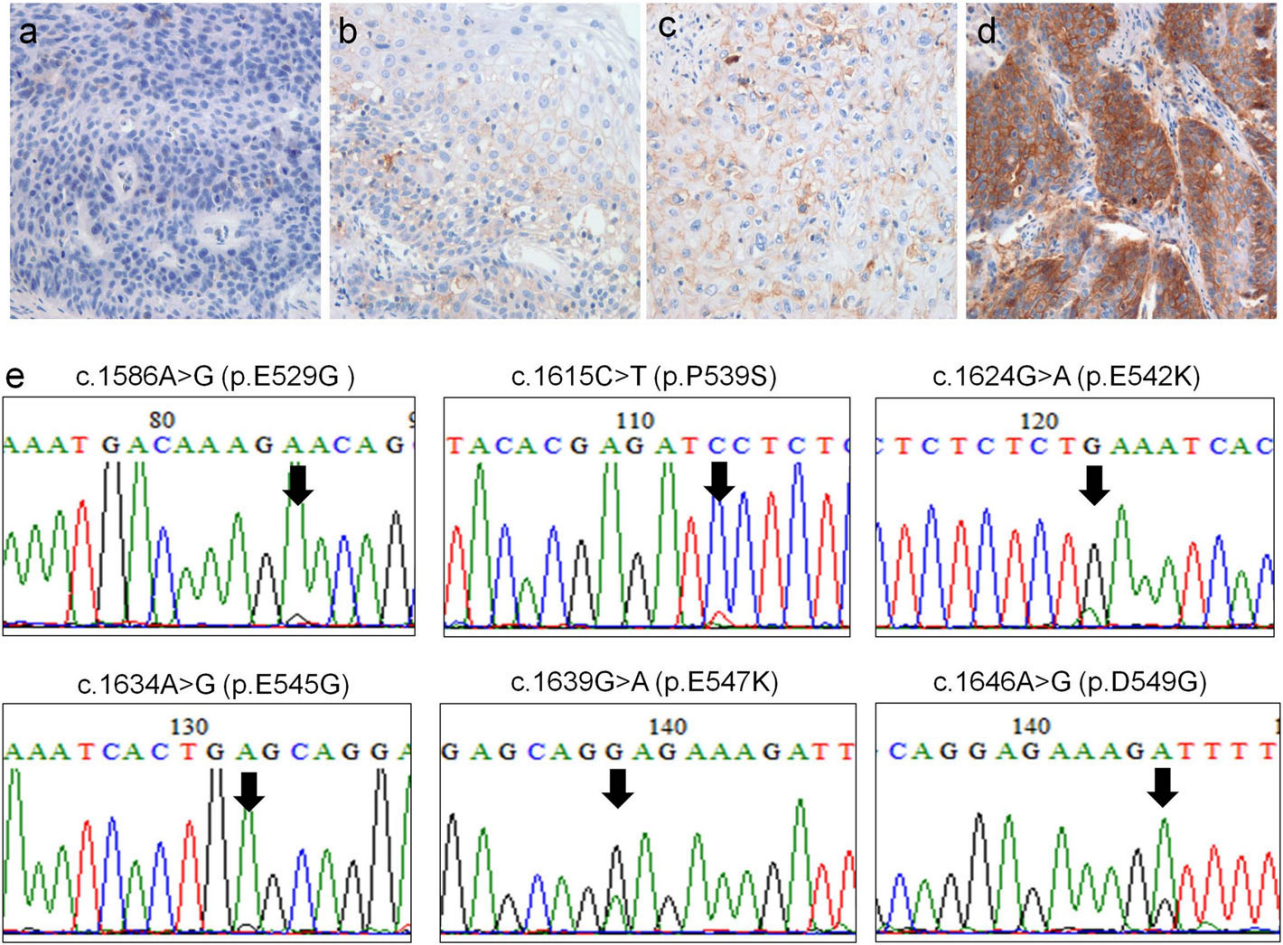
Representative immunohistochemical images of PD-L1 expression in esophageal squamous cell carcinoma. PD-L1 expression was scored from 0 (**a**), 1+ (**b**), 2+ (**c**), to 3+ (**d**). Cases with scores of 0 were considered PD-L1-negative, while cases with scores ≥1+ were considered positive for PD-L1 expression. (**e**) Electropherograms of the mutated sequences in exon 9 of *PIK3CA* gene. (Original magnification, × 200)

Twenty-three (35.9%) were PD-L1-positive [score 1+ (10.9%, *n* = 7), 2+ (10.9%, n = 7), and 3+ (14.1%, *n* = 9)] and 41 (64.1%) were PD-L1-negative (Fig. 1a-d). Patients with PD-L1-positive tumors were significantly correlated with only light smoking status (*P* = 0.048). Of the 8 ESCC tumor tissues (12.5%) with *PIK3CA* mutation, 2 (25.0%) were PD-L1-positive. Of the 11 ESCC tumor tissues (17.2%) with MSI/dMMR, 3 (27.3%) were PD-L1-positive. PD-L1-positive tumors were not correlated with either *PIK3CA* mutation or MSI/dMMR (*P* = 0.700 and *P* = 0.732, respectively). *PIK3CA* mutation was significantly associated with female patients and younger age (≤60 years) (*P* = 0.019 and *P* = 0.043, respectively). MSI/dMMR was more frequently detected in younger patients (≤60 years) and well-differentiated tumors (*P* = 0.039 and *P* = 0.039, respectively).

None of the tissue samples exhibited mutations in the *KRAS* and *BRAF* genes. *PIK3CA* mutations were detected exclusively in the exon 9 [p.E529G (*n* = 1), p.P539S (n = 1), p.E542K (*n* = 2), p.E545G (n = 1), p.E547K (n = 2), and p.D549G (n = 1)] but not in exon 20 (Fig. 1e).

**Fig. 2:**
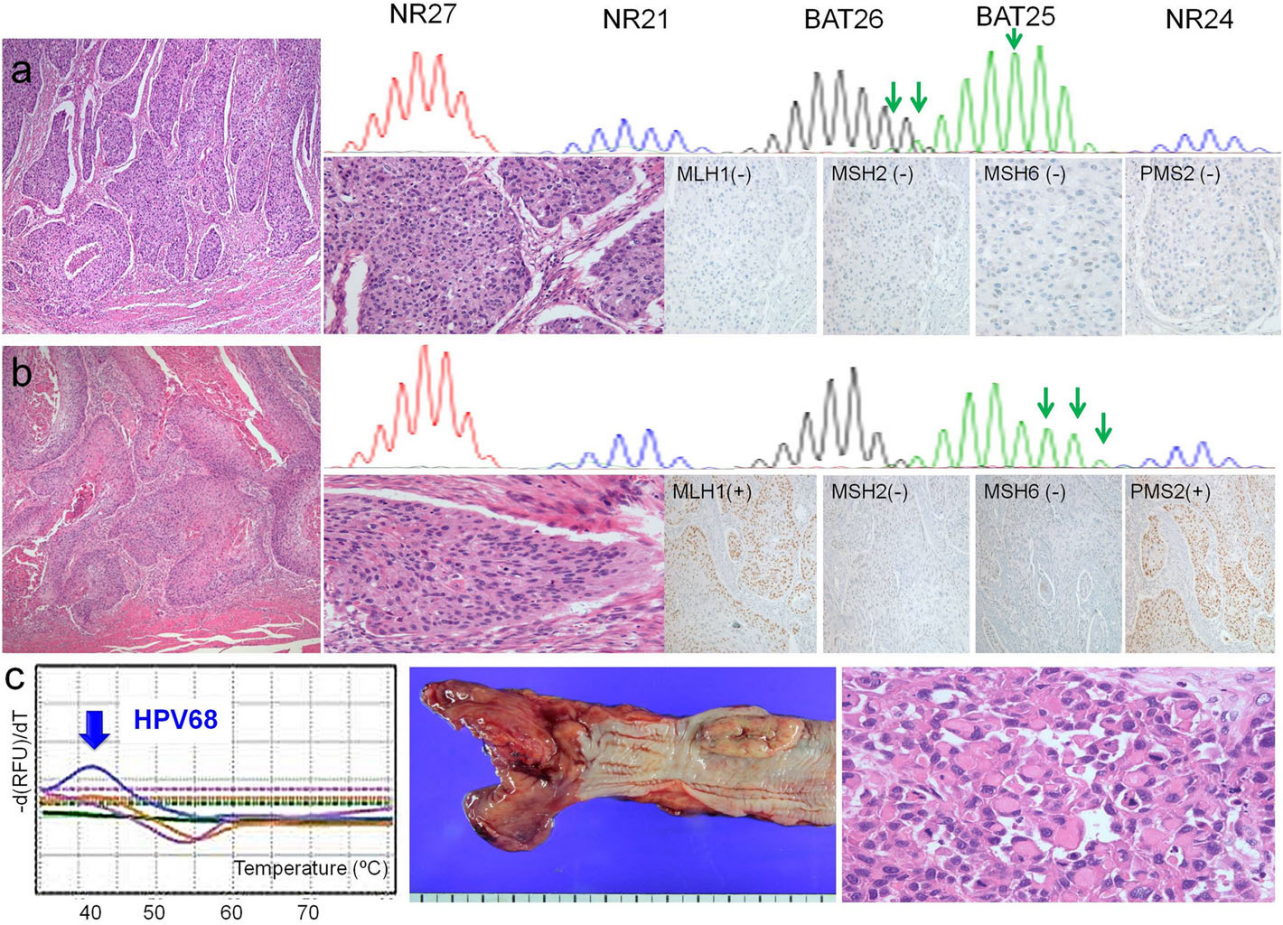
**a**-**b** Representative images of microsatellite instability-low and the corresponding immunohistochemical results of mismatch repair proteins and histological findings. (**a**) BAT25 marker showing microsatellite instability and lack of immunohistochemistry of MLH1, MSH2, MSH6, and PMS2 are noted in the poorly-differentiated squamous cell carcinoma with high tumor infiltrating lymphocytes. **b** BAT25 marker showing microsatellite instability and lack of immunohistochemistry of MSH2 and MSH6 are observed in the well-differentiated squamous cell carcinoma with low tumor infiltrating lymphocytes. **c** Real-time peptide nucleic acid probe-based fluorescence melting curve analysis reveals the HPV68 genotyping. A fungating mass (3.5 × 1.8 × 0.8 cm) is observed in the lower portion of esophagus. Poorly-differentiated esophageal squamous cell carcinoma with discohesive tumor cells

Among the 11 samples with dMMR, immunohistochemical analysis revealed that the expression of MSH2/MSH6, MLH1/MSH2/PMS2/MSH6, and MSH6 proteins was not detected in 7, 2, and 2 samples, respectively. However, dMMR cases exhibited only MSI-L with allelic size variation in one of the five mononucleotide markers. The remaining tumors were classified as MSS. None of the tumors exhibited MSI-H. Among the four quasi-monomorphic markers, only BAT25 marker exhibited MSI. Cases that did not exhibit MLH1, MSH2, MSH6, and PMS2 expression, which was evaluated by immunohistochemical analysis, exhibited poorly-differentiated squamous cell carcinoma with high TILs (Fig. 2a). The remaining cases lacking at least one MMR proteins exhibited well-differentiated squamous cell carcinomas with low TILs (Fig. 2b).

### HPV infection in esophageal cancers

Only one patient (1.6%) harbored high-risk HPV68 infection in the ESCC tissue, which exhibited PD-L1-negative status, wild-type *PIK3CA* gene expression, and MSS status (Fig. 2c). The patient was a 68-year-old male with stage IIIA cancer in the lower esophagus and lymph node metastasis. The patient was a never-alcohol drinker but a heavy smoker smoking 25 pack/year. The patient did not exhibit synchronous or metachronous malignancies. The analysis of H&E slide of this patient revealed a large, exophytic mass (3.5 × 1.8 cm) comprising poorly-differentiated tumor with markedly high TILs. The tumor cells were relatively non-cohesive. In addition to the main tumor mass, mild to moderate dysplasia with koilocytosis was observed. The patient did not receive any postoperative adjuvant therapy and was alive without any recurrence for 32 months.

### Clinicopathological correlation with survival

**Fig. 3:**
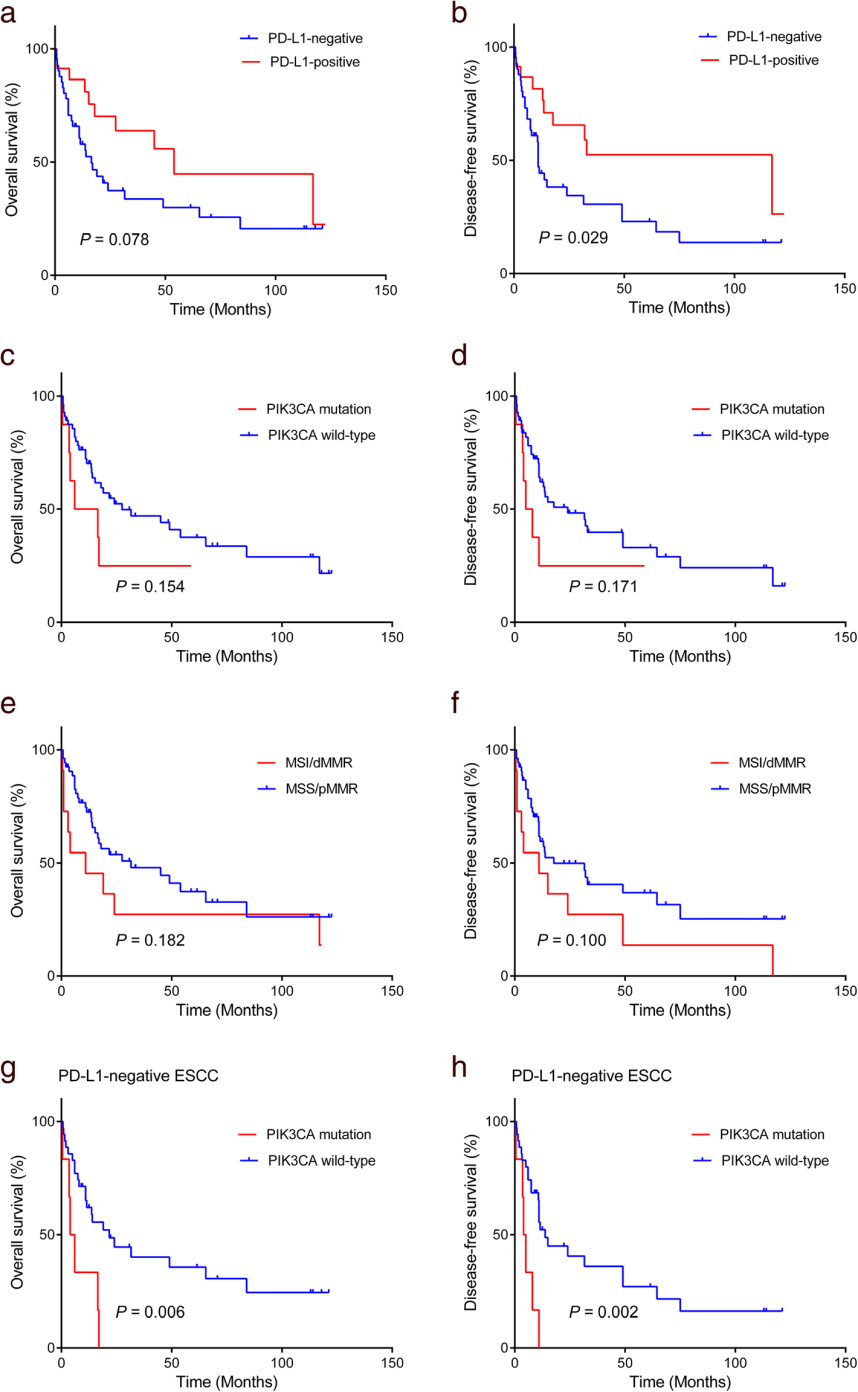
Kaplan–Meier survival curves according to PD-L1 expression (**a**, **b**), *PIK3CA* mutation (**c**, **d**), MSI/dMMR (**e**, **f**) for overall survival and disease-free survival

We performed Kaplan-Meier survival analyses to determine whether PD-L1 expression, *PIK3CA* mutation, and MSI/dMMR were correlated with OS or DFS in patients with ESCC. Kaplan-Meier curves revealed that the PD-L1-positive tumors exhibited a favorable OS when compared to the PD-L1-negative tumors (median 54 vs. 16.5 months) with borderline statistical significance (log-rank test: *P =* 0.078). Patients with PD-L1-positive tumors exhibited better DFS rate than those with PD-L1-negative tumors (median 117 vs. 11 months) (log-rank test: *P =* 0.029) (Fig. 3a-b).

There were significant differences in the OS (median 6 vs. 27 months) and DFS (median 5 vs. 24 months) between patients with *PIK3CA*-mutated and those with *PIK3CA*-wild-type tumors (log-rank test: *P =* 0.154 and *P =* 0.171, respectively) (Fig. 3c-d).

There were no significant differences in the OS (median 11 vs. 31.6 months) and DFS (median 11 vs. 17.7 months) between patients with MSI/dMMR and those with MSS/pMMR (log-rank test: *P =* 0.182 and *P =* 0.100, respectively) (Fig. 3e-f).

To investigate the prognostic factors for OS and DFS in patients with ESCC, univariate and multivariate Cox proportional hazard regression analyses were performed (Table 3). The univariate analysis revealed that vascular invasion was significantly associated with poor OS (*P* = 0.047). Additionally, PD-L1 positive status and the presence of dysplasia were significantly associated with DFS (*P* = 0.034 and *P* = 0.031, respectively). The multivariate analysis, including the variables of statistical or borderline association, revealed that PD-L1 positive status was an independent favorable prognostic factor for OS and DFS (*P* = 0.023, hazard ratio [HR] 0.416, 95% confidence interval [CI] 0.196–0.885; *P* = 0.014, HR 0.379, 95% CI 0.174–0.824).

**Table 3:** Univariate and multivaritate analyses of overall survival and disease-free survival of patients with ESCC

	Overall survival	*P*	Disease-free survival	*P*
	HR (95% CI)		HR (95% CI)	
Univariate analysis				
Gender (Male vs. female)	0.290 (0.040–2.124)	0.223	0.345 (0.047–2.517)	0.294
Age (y) (< 60 vs. ≥60)	0.786 (0.401–1.507)	0.469	0755 (0.401–1.423)	0.385
Alcohol (Light vs. Heavy)	1.711 (0.873–3.354)	0.118	1.825 (0.940–3.543)	0.075
Smoking (Light vs. Heavy)	1.261 (0.664–2.395)	0.479	1.239 (0.664–2.311)	0.501
Stage (I/II vs. III/IV)	1.421 (0.744–2.714)	0.288	1.559 (0.830–2.927)	0.167
Differentiation (WD vs. MD/PD)	1.425 (0.641–3.170)	0.385	1.418 (0.667–3.018)	0.364
Location (upper/middle vs. lower)	1.139 (0.560–2.316)	0.720	1.305 (0.658–2.591)	0.446
Adjuvant therapy	1.437 (0.698–2.960)	0.325	1.814 (0.916–3.593)	0.088
Resection margin (R0 vs. R1)	1.050 (0.370–2.976)	0.927	1.286 (0.454–3.638)	0.636
Vascular invasion	1.992 (1.009–3.934)	**0.047**	1.932 (0.992–3.765)	0.053
Lymphatic invasion	1.856 (0.944–3.648)	0.073	1.905 (0.989–3.671)	0.054
Perineural invasion	1.951 (0.934–4.073)	0.075	2.083 (0.998–4.345)	0.051
Dysplasia	0.574 (0.296–1.115)	0.101	0.478 (0.716–3.064)	**0.031**
PD-L1 (negative vs. positive)	0.084 (0.256–1.090)	0.078	0.458 (0.222–0.944)	**0.034**
MSI/dMMR	1.667 (0.780–3.564)	0.187	1.804 (0.879–3.704)	0.108
*PIK3CA* mutation	1.876 (0.777–4.530)	0.162	1.818 (0.756–4.373)	0.182
Multivariate analysis				
Vascular invasion	1.818 (0.824–4.009)	0.139	1.622 (0.726–3.623)	0.239
Lymphatic invasion	1.557 (0.707–3.429)	0.271	1.725 (0.747–3.983)	0.202
Perineural invasion	1.435(0.646–3.188)	0.376	1.069(0.449–2.544)	0.880
PD-L1 (negative vs. positive)	0.416 (0.196–0.885)	**0.023**	0.379 (0.174–0.824)	**0.014**
Alcohol (Light vs. Heavy)	–	–	1.792(0.868–3.701)	0.115
Adjuvant therapy	–	–	2.009(0.896–4.504)	0.090
Dysplasia	–	–	0.701(0.341–1.444)	0.336

*ESCC* esophageal squamous cell carcinoma, *HR* hazard ratio, *CI* 95% confidence interval, *WD* well-differentiation, *MD* moderately-differentiation, *PD* poorly-differentiation, *PD-L1* programmed death ligand-1, *MSI* microsatellite instability, *dMMR* deficient mismatch repair

### Prognostic impact of MSI/dMMR and *PIK3CA* mutation according to PD-L1 expression

Further, we analyzed the prognostic value of *PIK3CA* mutation and MSI/dMMR for OS and DFS according to PD-L1 expression.

In the PD-L1-negative esophageal cancers, patients with *PIK3CA*-mutated tumors exhibited poorer OS and DFS than patients with *PIK3CA*-wild-type tumors (*P* = 0.006 and *P* = 0.002, respectively) (Fig. 3g-h). However, there were no significant differences in the OS or DFS of patients with PD-L1-positive esophageal cancers with or without *PIK3CA* mutation (*P* = 0.239 and *P* = 0.227, respectively).

There were no significant differences in the OS or DFS for both PD-L1-positive and PD-L1-negative esophageal cancers with or without MSI/dMMR (*P* = 0.936 and *P* = 0.883 for PD-L1 positivity; *P* = 0.141 and *P* = 0.070 for PD-L1 negativity).

## Discussion

In this study, we screened 64 ESCC tissue specimens for PD-L1 expression, HPV infection, MSI/dMMR, and mutations in the *KRAS*, *BRAF*, or *PIK3CA* genes to determine their prognostic value in esophageal cancers. We confirmed that Korean ESCC cases were associated with male patients (94%) with a median age of 63 years, heavy smoking (54.7%) or alcohol drinking (50%), and poor 5-year OS (23.9%). The multivariate analysis revealed that PD-L1 expression, which was detected in one-third of ESCC cases, was an independent favorable prognostic factor for OS and DFS. The combined analysis revealed that *PIK3CA* mutation in the PD-L1-negative ESCCs was a poor prognostic factor for both OS and DFS. The incidence of HPV was very rare, while the *KRAS* and *BRAF* mutations were not detected in ESCCs. To the best of our knowledge, currently this is the first study evaluating combined the present druggable and targetable biomarkers in ESCCs.

Previously, several studies have reported that the frequencies of tumor cells exhibiting PD-L1 expression in ESCC range from 18.4 to 79.7%, which might be attributable to the differences in the antibodies used, interpretation criteria, specimen types affected by neoadjuvant therapy, and the geographic or racial characteristics [19–25]. In this study, PD-L1-positive tumors were observed in 35.9% of cases, which is highly consistent with a previous Korean population-based study (33.5%) [16]. As approximately one-third of the cells exhibited PD-L1 expression in ESCCs, PD-L1 can serve as prognostic and predictive biomarkers in esophageal cancer cases associated with limited chemotherapeutic options. To facilitate the clinical application of PD-L1, this study specifically utilized the SP142 anti-PD-L1 antibody, a U.S. FDA-approved antibody for a validated immunohistochemistry protocol, and the criterion of ≥5% of PD-L1, which were used for identifying patients with advanced urothelial cancers for atezolizumab treatment [26]. In the present study, PD-L1 positivity was an independent prognostic factor for favorable DFS in the patients with curatively resected, adjuvant therapy-naive ESCC. PD-L1 positivity also exhibited better OS than those with PD-L1 negativity in the multivariate analysis, although the univariate analysis did not reach statistical significance (*P* = 0.078), of which result might be confounded by the factors that included or excluded in the analyses for OS. Our finding relevant to favorable DFS concurred with that of previous studies that demonstrated favorable prognosis of PD-L1 expression in surgically resected ESCCs without preoperative or neoadjuvant treatment [22–24, 27]. In this study, high PD-L1 expression was prevalent among light or non-smoker patients without lymph node metastasis and with high TILs. This may be because the non-smoking or light smoking patients with PD-L1-positive tumors had highly immunogenic tumors [25], which indicated that a strong adaptive immune response may attack the tumor microenvironment and ultimately influence the favorable clinical outcomes of patients [11, 23]. Some studies have reported a poor prognostic correlation of PD-L1 expression in patients treated with neoadjuvant concurrent chemoradiotherapy [19, 20], while others have demonstrated no correlation between PD-L1 expression and survival in the patients undergoing postoperative chemotherapy [21]. The discrepancy in the prognostic values of PD-L1 may be because the neoadjuvant or adjuvant treatment agents may serve as confounders for the PD-1/PD-L1 axis and because the PD-L1 expression protects tumor cells from pro-apoptotic agents [10]. Lim et al. [19] also reported PD-L1 expression significantly increased after concurrent chemoradiation therapy. Likewise, Ng et al. [28] also described that PD-L1 expression was significantly induced in ESCC cell lines after standard chemotherapy treatments, suggesting that the potential benefit of combined conventional chemotherapy together with anti-PD-L1 immunotherapy to achieve better treatment outcome. Some studies have reported that the PD-1/PD-L1 axis mediates resistance to radiotherapy and anti-CTLA-4 antibody immunotherapy [29]. Therefore, an optimal treatment combination of immune checkpoint inhibitors and other therapies is required for the clinical treatment of patients with ESCC.

The *PIK3CA* mutation activates many downstream pathways that regulate critical cellular functions involved in the development of cancer [8]. In this study, *PIK3CA* mutation was detected in 12.5% of esophageal cancers and was associated with female or younger patients (≤60 years) lacking mutations in the *KRAS* and *BRAF* genes. There are limited studies that have evaluated *PIK3CA* mutation, which has an incidence of 1.5–21%, in esophageal cancers [30–33]. Additionally, mutations in the *KRAS* and *BRAF* genes are reported to be extremely rare (0.5–1%) in ESCCs [30, 34]. The detection of *PIK3CA* mutation in ESCCs may suggest that *PIK3CA* mutation may be involved in the development and progression of esophageal cancers, especially in female and younger patients (≤60 years) who are not commonly affected by ESCCs. Consistent with this finding, PI3K inhibitors are reported to inhibit the growth of an esophageal cancer cell line with *PIK3CA* mutation in vitro [32]. Additionally, mutations in the *KRAS*, *BRAF*, and *PIK3CA* genes have emerged as an important predictive marker of resistance to epidermal growth factor receptor (EGFR)-targeting monoclonal antibodies or tyrosine kinase inhibitors in colorectal and lung cancers [10]. However, the effectiveness of these therapies is unclear in esophageal cancers. In this study, we demonstrated that approximately one-tenth of ESCC cases exhibited *PIK3CA* mutation. However, none of the ESCC cases exhibited mutations in the *KRAS* and *BRAF* genes. This suggested that a subset of these patients may respond to PI3K inhibitors but may not respond to EGFR-targeting treatment.

The prognostic significance of *PIK3CA* mutation has been controversial. *PIK3CA* mutation has been associated with favorable prognosis in patients with curatively resected ESCCs in a Japanese population-based study [31], whereas a Korean population-based study reported no correlation between *PIK3CA* mutation and survival [33]. We also observed that *PIK3CA* mutation alone does not affect survival. However, the combined analyses revealed that the *PIK3CA* mutation in PD-L1-negative esophageal cancers was significantly correlated with poor survival. This indicated that *PIK3CA* mutation may be potentially important and may be an alternative prognostic biomarker in PD-L1-negative tumors that can be used to identify high-risk patients with worse clinical outcome who require more intensive therapy and follow-up.

There have been limited studies on MSI/dMMR in ESCC, of which results appear to different from conventional colorectal or gastric adenocarcinomas [17, 35, 36]. Previous studies have demonstrated that the frequency of MSI in ESCC varies from 2 to 66.7% [17, 35, 36]. MSI/dMMR in esophageal cancer may predict the clinical response to PD-L1 [13]. In this study, we detected MSI/dMMR in 17.2% of ESCCs, among which one-fourth was PD-L1-positive. We observed that MSI/dMMR represented younger patients (≤60 years) and well-differentiated tumors but did not have any survival impact in esophageal cancers. In this study, we evaluated the MSI/dMMR status using immunohistochemistry and quasi-monomorphic mononucleotide markers. We observed that the dMMR cases exhibited only MSI-L but not MSI-H. There was no perfect concordance between dMMR and MSI. Similarly, Hayashi et al. [17] reported that MSI at one or more loci was observed in 40% of ESCCs with only one classified as MSI-H. Additionally, the loss of MLH1 expression, which was evaluated by immunohistochemistry, did not correspond to MSI. The MSI and MMR protein expression patterns in the ESCCs seem to be different from those in the colorectal or gastric cancers. MSI-L observed in ESCC could result from defects in MMR proteins other than MSH2 and MLH1 or from defects in other genes not involved in MMR, or it could simply represent a background level of genetic instability that may be present in all tumors [17, 35]. The squamous cell histology in esophageal cancers might contribute to the discrepancy between MSI and dMMR because other intestinal MSI cancers are related to intestinal-type adenocarcinomas with mucinous and lymphoid-rich histology [17, 36].

In this study, only 1 tumor (1.6%) carried the high-risk HPV68 infection. This indicated that the HPV infection rate in ESCC samples is very low and that HPV is not the etiological cause of ESCC, which concurred with findings of a previous study [8]. Globally, there is high variability in the detection rates of HPV in ESCCs. The HPV detection rate in the tumor tissues in the high-ESCC-incidence countries is significantly higher than that in the low-ESCC-incidence countries (up to 64% in China vs. up to 17% in North America) [37]. In Korea, the incidence of esophageal cancers is low with 2457 new cases reported in 2017, which ranked 19 out of 23 malignancies [3]. The low prevalence of esophageal cancers in Korea may result in a low HPV detection rate in ESCCs. This is the first study investigating the incidence of HPV in ESCCs of Korea.

The limitations of this study include the retrospective analysis of archival samples, the relatively low number of samples, and the possible selection bias of current study. Considering the low incidence of esophageal cancers, the enrolled number of 64 patients is not small sample size. The results of this study provide valuable insights into the associations between surgically resected ESCCs and druggable targets to date.

## Conclusions

PD-L1 expression along with *PIK3CA* mutation and MSI/dMMR was detected in approximately one-third and one-fourth of ESCC cases, respectively, with no mutations detected in the *KRAS* and *BRAF* genes. The MSI/dMMR pattern in ESCCs was different from those in the colorectal or gastric cancers. The favorable prognostic impact of PD-L1 on ESCCs is clinically relevant in surgically resected patients and its combined analysis with *PIK3CA* mutations may alternatively identify high-risk patients with worse clinical outcomes in patients with PD-L1-negative tumors. This suggested that the therapeutic strategy of ESCC may be more complicated than other malignancies: the combination of immunotherapeutic strategies and molecular target therapy may be possibly applicable and the PD-L1 expression in PD-L1-postive ESCCs and *PIK3CA* mutation in PD-L1-negative ESCCs may be beneficial in curatively resected ESCCs.

## Supplementary information

**Additional file 1: Supplementary Table 1** Clinicopathologic characteristics of patients with ESCC and associations of smoking and alcohol consumptions.

## Data Availability

The datasets generated and /or analysed during the current study are not publicly available due the institutional review board restricts the use of the datasets to the current study only.

## Abbreviations

ESCC: Esophageal squamous cell carcinoma
PD-L1: Programmed death-ligand 1
MSI: Microsatellite instability
pMMR: patent mismatch repair
dMMR: deficient mismatch repair
HPV: Human papillomavirus
OS: Overall survival
DFS: Disease-free survival
AJCC: Amedican Joint Committee on Cancer 8th edition
TIL: Tumor-infiltrating lymphocyte
