# Supplementary material for: Significance of druggable targets (PD-L1, KRAS, BRAF, PIK3CA, MSI, and HPV) on curatively resected esophageal squamous cell carcinoma
Source: Diagn Pathol. 2020 Oct 14;15:126. doi: 10.1186/s13000-020-01045-4 (PMC7557072; doi:10.1186/s13000-020-01045-4)
Supplement: Supplementary file 1 — Additional file 1: Supplementary Table 1 Clinicopathologic characteristics of patients with ESCC and associations of smoking and alcohol consumptions. [file 13000_2020_1045_MOESM1_ESM.docx]

**Supplementary Table 1** Clinicopathologic characteristics of patients with ESCC and associations of smoking and alcohol consumptions

|  | Smoking | | *P* | Alcohol | | *P* |
| --- | --- | --- | --- | --- | --- | --- |
|  | Light  n = 34 (%) | Heavy  n = 30 (%) |  | Light  n = 26 (%) | Heavy  n = 38 (%) |  |
| Sex |  |  | 0.116 |  |  | **0.024** |
| Male | 30 (88.2) | 30 (100) |  | 22 (84.6) | 38 (100) |  |
| Female | 4 (11.8) | 0 (0.0) |  | 4 (15.4) | 0 (0.0) |  |
| Age (years) |  |  | 0.151 |  |  | 0.450 |
| ≤60 | 11 (32.4) | 15 (50.0) |  | 9 (34.6) | 17 (44.7) |  |
| >60 | 23 (67.6) | 15 (50.0) |  | 17 (65.4) | 21 (55.3) |  |
| Location |  |  | 0.412 |  |  | 0.784 |
| Upper/Middle | 22 (64.7) | 23 (76.7) |  | 19 (73.1) | 26 (68.4) |  |
| Lower | 12 (35.3) | 7 (23.3) |  | 7 (26.9) | 12 (31.6) |  |
| Differentiation |  |  | 0.075 |  |  | 0.450 |
| WD | 10 (29.4) | 16 (53.3) |  | 9 (34.6) | 17 (44.7) |  |
| MD/PD | 24 (70.6) | 14 (46.7) |  | 17 (65.4) | 21 (55.3) |  |
| T category |  |  | 0.207 |  |  | 0.124 |
| T1-T2 | 22 (64.7) | 14 (46.7) |  | 18 (69.2) | 18 (47.4) |  |
| T3-T4 | 12 (35.3) | 16 (53.3) |  | 8 (30.8) | 20 (52.6) |  |
| N category |  |  | **0.040** |  |  | 0.609 |
| N0 | 25 (73.5) | 14 (46.7) |  | 17 (65.4) | 22 (57.9) |  |
| N1-3 | 9 (26.5) | 16 (53.3) |  | 9 (34.6) | 16 (42.1) |  |
| AJCC stage |  |  | 0.071 |  |  | 0.795 |
| I-II | 25 (73.5) | 15 (50.0) |  | 17 (65.4) | 23 (60.5) |  |
| III-IV | 9 (26.5) | 15 (50.0) |  | 9 (34.6) | 15 (39.5) |  |
| Lymphatic invasion |  |  | 0.314 |  |  | 0.456 |
| Absent | 16 (47.1) | 10 (33.3) |  | 12 (46.2) | 14 (36.8) |  |
| Present | 18 (52.9) | 20 (66.7) |  | 14 (53.8) | 24 (63.2) |  |
| Vascular invasion |  |  | 0.736 |  |  | 1.000 |
| Absent | 24 (70.6) | 20 (66.7) |  | 18 (69.2) | 26 (68.4) |  |
| Present | 10 (29.4) | 10 (33.3) |  | 8 (30.8) | 12 (31.6) |  |
| Perineural invasion |  |  | 0.351 |  |  | 0.534 |
| Absent | 29 (85.3) | 22 (73.3) |  | 22 (84.6) | 29 (76.3) |  |
| Present | 5 (14.7) | 8 (26.7) |  | 4 (15.4) | 9 (23.7) |  |
| Skip lesion |  |  | 0.067 |  |  | 0.847 |
| Absent | 30 (88.2) | 20 (66.7) |  | 20 (76.9) | 30 (78.9) |  |
| Present | 4 (11.8) | 10 (33.3) |  | 6 (23.1) | 8 (21.1) |  |
| TIL density |  |  | **0.018** |  |  | 0.180 |
| Low | 7 (20.6) | 15 (50.0) |  | 6 (23.1) | 16 (42.1) |  |
| High | 27 (79.4) | 15 (50.0) |  | 20 (76.9) | 22 (57.9) |  |
| HPV |  |  | 0.469 |  |  | 0.406 |
| Negative | 34 (100) | 29 (96.7) |  | 25 (96.2) | 38 (100) |  |
| Positive | 0 (0.0) | 1 (3.3) |  | 1 (3.8) | 0 (0.0) |  |
| MSI status |  |  | 0.322 |  |  | 0.089 |
| MSS/pMMR | 30 (88.2) | 23 (76.7) |  | 24 (92.3) | 29 (76.3) |  |
| MSI/dMMR | 4 (11.8) | 7 (23.3) |  | 2 (7.7) | 9 (23.7) |  |
| *PIK3CA* gene |  |  | 0.850 |  |  | 0.564 |
| Wildtype | 30 (88.2) | 26 (86.7) |  | 22 (84.6) | 34 (89.5) |  |
| Mutated | 4 (11.8) | 4 (13.3) |  | 4 (15.4) | 4 (10.5) |  |
